# Association of walking speed with cognitive function in Chinese older adults: A nationally representative cohort study

**DOI:** 10.3389/fnagi.2022.1003896

**Published:** 2022-11-10

**Authors:** Jianping Liu, Kaiwang Cui, Qian Chen, Zhiteng Li, Jing Fu, Xiangwen Gong, Hui Xu

**Affiliations:** ^1^Department of Respiratory and Critical Care Medicine, Ganzhou Institute of Respiratory Diseases, The Fifth People’s Hospital of Ganzhou, Ganzhou, Jiangxi, China; ^2^Big Data Center, Beijing Children's Hospital, Capital Medical University, National Center for Children's Health, Beijing, China

**Keywords:** walking speed, cognitive function, older Chinese adults, national, cohort study

## Abstract

**Background:**

Slow walking speed has been shown to predict cognitive decline in older individuals, but studies conducted among Chinese older adults are scarce. We examined the association of walking speed with cognitive function and the trajectory of cognitive decline among Chinese adults aged 60 years and older.

**Methods:**

Data was from the China Health and Retirement Longitudinal Study (CHARLS), an ongoing nationally representative prospective cohort study. Walking speed was evaluated over a straight 2.5-meter flat course at baseline and categorized into tertiles (the lowest, middle, and highest). Cognitive function was assessed at each wave in three domains: episodic memory, mental status, and global cognition. Data were analyzed using linear mixed-effects models.

**Results:**

A total of 3,954 older adults (48.6% female; mean age: 67.6 ± 5.55 years) were followed for up to 7 years. Participants with lowest walking speed have poorer episodic memory (*β* = −0.37; 95% CI: −0.46, −0.28), mental status (*β* = −0.45; 95% CI: −0.60, −0.29), and global cognition (*β* = −0.81; 95% CI: −1.03, −0.60) over the follow-up. Compared with the highest tertile of walking speed, the lowest walking speed was associated with a faster decline in episodic memory (*β* = −0.04; 95% CI: −0.07, −0.02), mental status (*β* = −0.04; 95% CI: −0.07, −0.01), and global cognition (*β* = −0.06; 95% CI: −0.11, −0.01).

**Conclusion:**

Slower walking speed is associated with subsequent risk of poorer cognitive function and faster cognitive decline in older Chinese adults.

## Introduction

Population aging is becoming more serious worldwide, and the number of people aged 60 years and above will be more than 2 billion in 2050 according to the World Health Organization (Organization, 2015). Meanwhile, cognitive impairment is one of the most common conditions in old adults (Langa and Levine, 2014). It was reported that the prevalence of mild cognitive impairment in people aged ≥50 years ranged from 6.1 to 30.4% in low-and middle-income countries (McGrattan et al., 2021), and 15.4% of the elderly over the age of 55 have cognitive impairment in China (Deng et al., 2021). Cognitive impairment could result in significant disability and dependence in older adults, and challenge health- and social-care systems (Oba et al., 2021). Therefore, identifying markers that predict cognitive impairment is a major subject of current interest (Li and Zhang, 2015; Montero-Odasso et al., 2019).

Some risk prediction models for cognitive impairment have been developed up to now (Davis et al., 2018; Hu et al., 2021; Palmqvist et al., 2021). However, current prediction models have shown low to moderate predictive ability with large variability, and exploring new markers is necessary (Kikkert et al., 2016). Motor function is possibly candidate indicator for predicting cognitive impairment or decline, of which walking speed is one of the most competitive markers (Grande et al., 2019; Rosso et al., 2019). Some previous cross-sectional and longitudinal studies suggested that lower walking speed was associated with cognitive decline (Hooghiemstra et al., 2017; Chou et al., 2019; Knapstad et al., 2019), but some others did not find the association (Atkinson et al., 2010; Donoghue et al., 2018). Up to now, the research on the prospective relationship between walking speed and cognitive impairment in Chinese adults is still scarce.

In the present study, we aimed to verify the hypothesis that low walking speed was associated with poor cognition function and faster rates of cognitive decline among elderly Chinese using data from the China Health and Retirement Longitudinal Study (CHARLS).

## Methods

### Study population

The study population was derived from the CHARLS, a Chinese nationwide longitudinal survey conducted by the National School of Development at Peking University on people aged 45 years or above. Details of the study design and the evaluation protocol regarding the CHARLS have been provided previously (Zhao et al., 2014). In brief, participants were recruited from 450 communities and administrative villages distributed in 28 provinces of China through multistage probability sampling, and these participants were followed every 2 or 3 years. The data of the CHARLS cohort were available for the baseline survey in 2011 (wave 1), the first follow-up survey in 2013 (wave 2), the second follow-up survey in 2015 (wave 3), and the third follow-up survey in 2018 (wave 4). At the time of enrollment and thereafter, each participant underwent a uniformly structured questionnaire (including demographics, health status, functioning, and so on) and anthropometrics measurements (e.g., height, weight, and walking speed). The CHARLS was approved by the Biomedical Ethics Review Committee of Peking University (IRB00001052-11015). All participants signed the informed consent and repository consent that allowed their data to be shared after a detailed presentation of the risks and benefits associated with study participation.

A total of 17,708 participants aged ≥45 years were recruited at baseline in 2011–2012, and 13,978 individuals (78.9%) completed anthropometric and physical measurements. However, only 7,082 (39.9%) individuals aged 60 years and over were eligible for the walking speed test (Zhao et al., 2014). Of the 7,082 individuals, 2,484 at baseline were excluded including 236 with physical or mental disability, 156 with memory-related disease (e.g., Alzheimer’s disease, brain atrophy, and Parkinson’s disease), 172 with cancer, and 1942 with missing information about walking speed tests, and 622 having no information of cognitive function at baseline or during follow-up were also excluded. Thus, 3,954 participants were available for the current study (Supplementary Figure 1).

### Assessment of walking speed

All participants aged 60 years and above were eligible for the test. However, participants who had health problems (e.g., hip fracture) that prevented them from walking were excluded from the walking speed test. A 2.5-meter flat, the straight course was prepared and marked out with tape. Participants were asked to walk on the walkway at their usual speed. Walking aids such as canes and walkers were allowed to use if required. The time participants took to walk completely across the walkway was measured by using a stopwatch. The test was performed twice, and the average time of the two trials was converted to walking speed (m/s). The walking speed was further classified into tertiles as highest (≥ 0.72 m/s), middle (0.56–0.72 m/s), and lowest (≤ 0.56 m/s) according to the data from the current population. That is, the cut-points (0.56 m/s and 0.72 m/s) were defined as the 33rd and 66th percentile of walking speed.

### Assessment of cognitive function

Cognitive function was assessed at baseline and during the follow-up, and cognitive measurements used in CHARLS were adapted from the Health and Retirement Study (McArdle et al., 2007). According to the previous CHARLS studies (Li et al., 2020; Luo et al., 2021), we constructed three measures of cognitive function: episodic memory, mental status, and global cognition. Episodic memory was assessed through immediate and delayed word recall tests. The participants were asked to recall words as many as possible in any order immediately after the interviewer reads a list of 10 Chinese words (immediate recall). About 4 to 10 min later, the participants were instructed to recall the same list of words (delay recall). The score of episodic memory was calculated as the average number of words correctly recalled in the immediate and delayed recall tests, ranging from 0 to 10. Mental status was assessed through Telephone Interview of Cognitive Status (TICS) and figure drawing test. The TICS was a well-established measure to capture the intactness or mental status of individuals and consisted of 10 questions with a range of 0 to 10, including today’s date (month, day, and year), the day of the week and the season of the year, and serial 7 subtraction from 100 (up to five times). The figure drawing test was designed to measure visuospatial abilities by asking participants to draw a picture of two overlapping pentagons which have been shown earlier. Participants who successfully reproduced a similar picture received 1 point, and those who failed received 0 points. The score of mental status was calculated by adding the sum scores from TICS and the figure drawing test, ranging from 0 to 11. Global cognition was defined as the total score of episodic memory and mental status with a range of 0 to 21, and a higher score indicated better cognitive function.

As prevent literature reported (Levy, 1994; Hu et al., 2022), cognitive impairment was defined as 1 standard deviation (SD) below the mean value of global cognition for the age- appropriate population. Participants aged ≥60 years were grouped for every 5 years of age.

### Assessment of potential confounders

Data of covariates including age, sex, education, marital status, body mass index (BMI), smoking status, drinking status, hypertension, heart disease, stroke, diabetes, lung disease, and depressive symptoms were collected at baseline. Education was categorized into two groups: primary school and below or middle school and above. Marital status was defined as either married or unmarried. BMI was calculated as weight in kilograms divided by height in meters squared (kg/m^2^). Smoking and drinking were categorized as never, former, and current groups. Chronic diseases including hypertension, heart disease, stroke, diabetes, and lung disease were self-reported and dichotomized as yes and no.

### Statistical analysis

Differences in characteristics among the three groups of participants with the lowest, middle, and highest walking speed were evaluated using one-way analysis of variance or Kruskal–Wallis test for continuous variables, and Chi-square tests for categorical variables.

The linear mixed-effects models were used to analyze the associations of baseline walking speed with subsequent cognitive function. The linear -mixed effect models were first adjusted for age, sex, and education, follow-up time, and then additionally adjusted for potential confounders including BMI, smoking, alcohol consumption, hypertension, diabetes, stroke, heart disease, and lung disease.

To further examine the associations between baseline walking speed and the rate of cognitive decline, we added an interaction term of baseline walking speed and follow-up time into linear -mixed models with adjustment for potential confounders.

In the sensitivity analysis, multiple imputation by chained equation was used to impute data for 93 participants with missing data of covariates including age, education, BMI, smoking status, alcohol consumption, hypertension, heart disease, stroke, and lung disease. Furthermore, we repeated the models after we excluded 1,825 participants with arthritis (*n* = 1,260), hip fracture (*n* = 36), aid used (*n* = 11), cognitive impairment (*n* = 501), and psychiatric disease (*n* = 17) at baseline. The effect modification of sex or education in the associations between walking speed and the rate of cognitive decline was further explored, and the interaction term between sex or education and walking speed was added in the models first; then, stratified analysis by sex or education was also performed. In addition, the association between motoric cognitive risk syndrome (MCR) and cognitive function was also explored. MCR was defined as cognitive complaint and slow walking speed without dementia or impaired mobility, according to previously established protocol (Bai et al., 2021). The cognitive complaint was obtained by asking participants a self-reported question: “How would you rate your memory at present?” Respondents were identified as having cognitive complaint if answers were fair or poor, while not if answers were excellent, very good, or good. Slow walking speed was defined as 1 SD below the mean value of walking speed for the age- and sex- appropriate population.

All analyses were performed with Stata SE, version 15.0 (Stata Corp LP., College Station, Texas, and USA), and statistical significance was defined as two-tailed *p* values less than 0.05.

## Results

### Characteristics of the study population

A total of 3,954 participants (48.66% female; mean age: 67.59 ± 5.56 years) were included in the final analyses. Baseline walking speed were ranged from 0.31 to 1.24 m/s (mean = 0.66 m/s, SD = 0.20 m/s). The median [interquartile range (IQR)] of three cognitive domains (episodic memory, mental status, and global cognition) at baseline were 3 (2 to 4.5), 9 (6 to 10), and 12 (9.5 to 14.5), respectively. Compared with participants who had the highest walking speed, those with the lowest were more likely to be older, female and smoker, to have lower education, less, and physical activity, higher alcohol consumption, and more hypertension, stroke, and to have lower scores of episodic memory, mental status, global cognition at baseline (Table 1).

**Table 1:** Baseline characteristics of the study population by walking speed categories (*n* = 3,954).

Characteristics	Walking speed (in tertiles)			
	Highest *n* = 1,337 (33.81%)	Middle *n* = 1,316 (33.28%)	Lowest *n* = 1,301 (32.90%)	*p*
Age (years)	66.4 ± 4.84	67.6 ± 5.55	68.7 ± 5.97	<0.001
Female	533 (39.87)	659 (50.08)	732 (56.26)	<0.001
Education level				<0.001
Primary school and below	1,024 (76.65)	1,097 (83.36)	1,128 (86.70)	
Middle school and above	312 (23.35)	219 (16.64)	173 (13.30)	
BMI	23.0 ± 3.57	22.9 ± 3.78	22.9 ± 3.96	0.200
Smoking status				<0.001
Never	679 (50.86)	750 (57.08)	803 (61.82)	
Ever smoker	171 (12.81)	138 (10.50)	133 (10.24)	
Current smoker	485 (36.33)	426 (32.42)	363 (27.94)	
Alcohol consumption				<0.001
Never drinker	677 (50.64)	752 (57.32)	803 (61.72)	
Former drinker	169 (12.64)	136 (10.37)	147 (11.30)	
Current drinker	491 (36.72)	424 (32.32)	351 (26.98)	
Hypertension	380 (28.49)	377 (28.71)	415 (32.07)	0.081
Diabetes	81 (6.08)	91 (6.95)	82 (6.34)	0.650
Heart disease	174 (13.05)	200 (15.30)	180 (13.88)	0.244
Stroke	19 (1.42)	27 (2.05)	46 (3.54)	0.001
Lung disease	176 (13.18)	189 (14.43)	176 (13.54)	0.633
Episodic memory	3.5 (2.5–4.5)	3 (2–4)	3 (2–4)	<0.001
Mental status	9 (7–11)	9 (6–10)	8 (6–10)	<0.001
Global cognition	13 (10.5–14.5)	12 (9–14)	11.5 (8.5–14)	<0.001

Data are presented as mean ± standard deviations, median (interquartile range), or number (proportion %).

Missing data: Smoking status = 6, Alcohol consumption = 4, Hypertension = 13, Heart disease = 17, Stroke = 4, Lung disease = 9.

BMI, Body mass index.

### Association between baseline walking speed and subsequent cognitive function

The mean follow-up time was 5.77 years (SD = 1.73 years), ranging from 2.0 to 7.0 years. Table 2 shows the relationship of baseline walking speed with subsequent cognitive function through multi-adjusted linear mixed -effect models. Compared to the highest tertile of walking speed, the lowest tertile was associated with the worst episodic memory (*β* = −0.37; 95% CI: −0.46, −0.28), mental status (*β* = −0.45; 95%CI: −0.60, −0.29) and global function (*β* = −0.81; 95%CI: −1.03, −0.60). As the walking speed decreased, episodic memory, mental status, and global function showed a declining trend (*P* for trend <0.001 for three).

**Table 2:** The association between walking speed and subsequent cognitive function in the CHARLS.

Cognitive function	Walking speed	*β* (95% CIs)^a^	*p*	*β* (95% CIs)^b^	*p*
**Episodic memory**
	Highest	Reference		Reference	
	Middle	−0.24 (−0.32, −0.15)	<0.001	−0.22 (−0.31, −0.14)	<0.001
	Lowest	−0.37 (−0.46, −0.28)	<0.001	−0.37 (−0.46, −0.28)	<0.001
	*p for trend*	<0.001		<0.001	
**Mental status**					
	Highest	Reference		Reference	
	Middle	−0.21 (−0.36, −0.07)	0.005	−0.19 (−0.34, −0.04)	0.011
	Lowest	−0.45 (−0.60, −0.29)	<0.001	−0.45 (−0.60, −0.29)	<0.001
	*p for trend*	<0.001		<0.001	
**Global cognition**
	Highest	Reference		Reference	
	Middle	−0.48 (−0.68, −0.27)	<0.001	−0.45 (−0.65, −0.24)	<0.001
	Lowest	−0.82 (−1.03, −0.60)	<0.001	−0.81 (−1.03, −0.60)	<0.001
	*p for trend*	<0.001		<0.001	

a Adjusted for age, sex, education, and follow-up time.

b Adjusted for age, sex, education, BMI, smoking, alcohol consumption, hypertension, diabetes, stroke, heart disease, lung disease, and follow-up time. Abbreviations: CIs, confidence intervals; BMI, body mass index.

### Association between baseline walking speed and trajectories of cognitive decline

**Figure 1:**
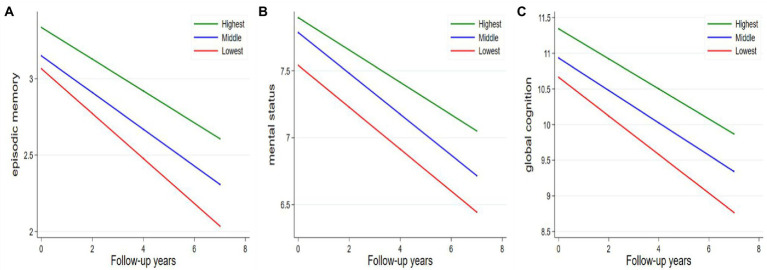
Average annual changes in cognitive function according to tertiles of walking speed. **(A)** Episodic memory; **(B)** mental status; **(C)** global cognition. Models were adjusted for age, sex, education, BMI, smoking, alcohol consumption, hypertension, diabetes, stroke, heart disease, lung disease, and follow-up time.

As indicated by the interaction between baseline walking speed and follow-up time, participants with slow walking speed showed a faster decline in episodic memory, mental status, and global function. The rates of decline in episodic memory, mental status, and global function among participants with the lowest walking speed were − 0.04 (*β* = −0.04; 95% CI: −0.07, −0.07), −0.04 (*β* = −0.04; 95% CI: −0.07, −0.01) and − 0.06 (*β* = −0.06; 95% CI: −0.11, −0.01) units per year faster than those with the highest walking speed, respectively **(** Table 3; Figure 1). Figure 1 depicts the trajectories of cognitive decline in different walking speed levels from the final linear -mixed effect model, focusing on the variance in the predicted slopes.

**Table 3:** The association between FGCRS and change in cognitive function over time in the CHARLS.

Cognitive function	Walking speed	*β* (95% CIs)^a^	*p*	*β* (95% CIs)^b^	*p*
**Episodic memory**
	Highest*time	Reference		Reference	
	Middle*time	−0.02 (−0.04, 0.01)	0.199	−0.02 (−0.04, 0.01)	0.209
	Lowest*time	−0.05 (−0.07, −0.02)	<0.001	−0.04 (−0.07, −0.02)	0.001
	*P for trend*	<0.001		0.001	
**Mental status**
	Highest*time	Reference		Reference	
	Middle*time	−0.03 (−0.06, 0.01)	0.104	−0.03 (−0.06, 0.01)	0.054
	Lowest*time	−0.04 (−0.07, −0.01)	0.029	−0.04 (−0.07, −0.01)	0.038
	*P for trend*	0.025		0.031	
**Global cognition**
	Highest*time	Reference		Reference	
	Middle*time	−0.01 (−0.06, 0.04)	0.611	−0.02 (−0.06, 0.03)	0.502
	Lowest*time	−0.07 (−0.12, −0.02)	0.009	−0.06 (−0.11, −0.01)	0.019
	*P for trend*	0.011		0.022	

a Adjusted for age, sex, education, and follow-up time.

b Adjusted for age, sex, education, BMI, smoking, alcohol consumption, hypertension, diabetes, stroke, heart disease, lung disease, and follow-up time. Abbreviations: CIs, confidence intervals; BMI, body mass index.

### Sensitivity analysis

The results were not altered much compared with those from the initial analyses when missing data were imputed by multiple imputation (Supplementary Tables 1, 2) and when we excluded 1,825 participants with arthritis, hip fracture, aid used, or psychiatric disease at baseline (Supplementary Tables 3, 4). Interaction between walking speed and sex or education on subsequent mental status was detected (*p* for interaction = 0.042 for sex; *p* for interaction = 0.030 for education). In the stratified analysis by sex, the association between lower walking speed and poorer performance in mental status was stronger in females and those with lower education (Supplementary Tables 5, 6). MCR at baseline was associated with subsequent worse cognitive performance, but not with the rates of cognitive decline in the three domains (Supplementary Tables 7, 8).

## Discussion

In this nationally prospective cohort study, we demonstrated the positively longitudinal association between walking speed and cognitive function among Chinese adults aged 60 years and older. We found that slower baseline walking speed was associated with subsequent poorer cognitive function including episodic memory, mental status, and global cognition. In addition, older Chinese adults with slower walking speeds had slightly faster rates of decline in cognitive function than those with higher walking speeds.

Walking speed appears one of the most convenient ways to measure motor function in older adults. Faster walking speed may be associated with younger biological age (Dempsey et al., 2022) and several better health-related outcomes (Studenski et al., 2011; Middleton et al., 2015; Yates et al., 2017). The relationship between walking speed and cognitive function has been exported in previous studies, but there was a lack of consensus in their findings(Atkinson et al., 2010; Hooghiemstra et al., 2017; Donoghue et al., 2018; Chou et al., 2019; Knapstad et al., 2019). One reason for the inconsistent results could be that the study population was from different countries.

Previous cross-section studies have investigated the association between walking speed and cognitive function in the Chinese general or special population. In 956 relatively healthy old Chinese aged ≥65 years, Liu et al. (2021) found faster 4-m walking speed was associated with better overall cognition and various cognitive domains, such as time orientation, place orientation, attention, and so on (Liu et al., 2021). Another study also found rapid gait speed was positively associated with verbal recall, verbal fluency, and overall cognition in general people aged 50 and above in Shanghai, China (Ruan et al., 2020). In addition, in the Chinese population aged 45 years or older with hypertension, faster-walking speed was related to better episodic memory and overall cognitive function (Zuo et al., 2019). Among middle-aged and older Chinese adults with diabetes, walking speed was significantly associated with figure drawing, one item of mental status (Zhang et al., 2019). However, no study focused on the longitudinal relationship in older Chinese. To the best of our knowledge, our study was the first to evaluate the longitudinal association of walking speed with subsequent cognition and the rate of decline in cognition in mainland China using a national cohort study. Thus, the elder population should not only focus on the daily step count but also a relatively quicker walking speed. Further evaluation of cognitive function is necessary once their walking speed is far beneath the average walking speed of normal elder adults.

As the walking or gait speed alone might lack specificity, future researches need to examine the association between the other more comprehensive gait parameters including dynamic metric of gait (e.g., gait variability, regularity, and stability) and cognitive function. Besides, high-quality randomized controlled trial is necessary to prove the effect of gait on cognition. Several mechanisms may account for the link between walking speed and the risk of poorer cognitive function and slightly faster cognitive decline. First, neurodegeneration not only contributes to cognitive decline (Cedres et al., 2021) but also to some extent affects walking abilities (Tian et al., 2016). Some evidence has indicated that slow walking speed was associated with higher levels of amyloid β in key brain areas(Del Campo et al., 2016) as well as the changes in subcortical white matter and cortical gray matter volumes (Holtzer et al., 2014). Second, Vascular burden is a possible underlying mechanism linking walking speed and cognitive function. A higher vascular burden hurts both cognitive function and walking abilities with white matter changes (Willey et al., 2013; Song et al., 2020). Third, Metabolic disorder may play a role in cognitive decline (Assuncao et al., 2018) and walking pace (Fredman et al., 2010). For example, insulin resistance could accelerate cognitive impairment with reduced beta-amyloid clearance and plaque development (Craft et al., 2013), and also lead to slow walking speed vie increasing the risk of frailty and Sarcopenia (Jang, 2016; Perez-Tasigchana et al., 2017). Fourth, another potential mechanism is the low-grade pro-inflammatory status which is typical of the aging process even in healthy older individuals (Ferrucci and Fabbri, 2018). High concentrations of inflammatory markers such as interleukin-6 levels may correlate with poor gait speed performance (Verghese et al., 2011) and more severe cognitive impairment (Lai et al., 2017). In addition, the inflammatory process is deleterious to the skeletal muscle which may result in mobility impairment (Reid et al., 2009).

The remarkable advantage of this study is the use of a national cohort with a relatively long follow-up, the repeated measurement of outcome (i.e., episodic memory, mental status, and global cognition), and the large sample size. However, several limitations in this study should be pointed out. First, cognitive function was examined using self-reported screening scales and divided into limited cognitive domains rather than specific cognitive domains. However, the self-reported screening scales still included three domains: episodic memory, mental status, and global cognition, and the measurement of cognition was reliable, informational, convenient and widely used. Second, 622 (13.5%) participants were lost during the follow-up time, which may influence the association between walking speed and cognitive function as determined in this study. Third, some potential confounders, such as cancer, heart disease, and lung disease, were assessed based on retrospective self-report, which could be subject to recall bias. Furthermore, residual confounding such as mental state and physical activity could not be completely ruled out.

In conclusion, based on a national prospective study, we provided evidence that slower walking speed is associated with a subsequent risk of poorer cognitive function and faster cognitive decline in older Chinese adults. Given it is a simple, inexpensive, and non-invasive measurement, walking speed holds potential as a pragmatic target for interventions for cognitive decline.

## Data availability statement

The datasets presented in this study can be found in online repositories. The names of the repository/repositories and accession number(s) can be found at: https://charls.charlsdata.com/pages/data/111/zh-cn.html.

## Ethics statement

The studies involving human participants were reviewed and approved by Biomedical Ethics Review Committee of Peking University. The patients/participants provided their written informed consent to participate in this study.

## Author contributions

HX and XG: full access to all the data in the study and take responsibility for the integrity of the data and the accuracy of the data analysis, study concept and design, and supervision. JL, KC, QC, ZL, and JF: acquisition, analysis, or interpretation of data. JL and KC: drafting of the manuscript. JL, KC, QC, ZL, JF, XG, and HX: critical revision of the manuscript for important intellectual content. KC: statistical analysis. JL, KC, HX, and XG: administrative, technical, or material support. All authors contributed to the article and approved the submitted version.

## Conflict of interest

The authors declare that the research was conducted in the absence of any commercial or financial relationships that could be construed as a potential conflict of interest.

## Publisher’s note

All claims expressed in this article are solely those of the authors and do not necessarily represent those of their affiliated organizations, or those of the publisher, the editors and the reviewers. Any product that may be evaluated in this article, or claim that may be made by its manufacturer, is not guaranteed or endorsed by the publisher.
