# Supplementary material for: Association of walking speed with cognitive function in Chinese older adults: A nationally representative cohort study
Source: Front Aging Neurosci. 2022 Nov 10;14:1003896. doi: 10.3389/fnagi.2022.1003896 (PMC9685315; doi:10.3389/fnagi.2022.1003896)
Supplement: Supplementary file 1 [file Data_Sheet_1.docx]

7082 participants aged 60 years and over were eligible for walking speed test.

2484 participants with disability (n=236), memory-related disease (n=156) or cancer (n=172) at baseline, and missing data of walking speed (n=1942) at baseline were excluded

Participants in final analysis, n=3954

Original sample, n=17708

Participants with baseline walking speed tests were interviewed

at baseline, n=4598

622 participants lost to follow-up

**Supplementary Figure 1.** Flowchart of study participants.

.

**Supplementary Table 1.** The association between walking speed and cognitive function at baseline in the CHARLS with multiple imputation for missing data.

| **Cognitive function** | **Walking speed** | **β (95% CIs) ^a^** | ***P*** |
| --- | --- | --- | --- |
| **Episodic memory** |  |  |  |
|  | **Highest** | Reference |  |
|  | **Middle** | -0.23 (-0.32, -0.14) | <0.001 |
|  | **Lowest** | -0.37 (-0.46, -0.28) | <0.001 |
|  | ***P* *for trend*** | <0.001 |  |
| **Mental status** |  |  |  |
|  | **Highest** | Reference |  |
|  | **Middle** | -0.21 (-0.35, -0.06) | 0.006 |
|  | **Lowest** | -0.45 (-0.60, -0.30) | <0.001 |
|  | ***P* *for trend*** | <0.001 |  |
| **Global cognition** |  |  |  |
|  | **Highest** | Reference |  |
|  | **Middle** | -0.46 (-0.67, -0.26) | <0.001 |
|  | **Lowest** | -0.82 (-1.03, -0.61) | <0.001 |
|  | ***P* *for trend*** | <0.001 |  |

^a^ Adjusted for age, sex, education, BMI, smoking, alcohol consumption, hypertension, diabetes, stroke, heart disease, lung disease, and follow-up time. Abbreviations: CIs, confidence intervals; BMI, body mass index.

**Supplementary Table 2.** The association between walking speed and change in cognitive function over time in the CHARLS with multiple imputation for missing data.

| **Cognitive function** | **Walking speed** | ***β* (95% CIs) ^a^** | ***P*** |
| --- | --- | --- | --- |
| **Episodic memory** |  |  |  |
|  | **Highest*time** | Reference |  |
|  | **Middle*time** | -0.01(-0.04, 0.03) | 0.709 |
|  | **Lowest*time** | -0.05(-0.08, -0.02) | 0.001 |
|  | ***P* *for trend*** | 0.001 |  |
| **Mental status** |  |  |  |
|  | **Highest*time** | Reference |  |
|  | **Middle*time** | -0.02(-0.07, 0.03) | 0.381 |
|  | **Lowest*time** | -0.03(-0.08, -0.01) | 0.032 |
|  | ***P* *for trend*** | 0.043 |  |
| **Global cognition** |  |  |  |
|  | **Highest*time** | Reference |  |
|  | **Middle*time** | -0.01 (-0.07, 0.06) | 0.831 |
|  | **Lowest*time** | -0.07 (-0.14, -0.01) | 0.039 |
|  | ***P* *for trend*** | 0.037 |  |

^a^ Adjusted for age, sex, education, BMI, smoking, alcohol consumption, hypertension, diabetes, stroke, heart disease, lung disease, and follow-up time. Abbreviations: CIs, confidence intervals; BMI, body mass index.

**Supplementary Table 3.** The association between walking speed and subsequent cognitive function in the CHARLS (n=2103).

| **Cognitive function** | **Walking speed** | **β (95% CIs) ^a^** | ***P*** |
| --- | --- | --- | --- |
| **Episodic memory** |  |  |  |
|  | **Highest** | Reference |  |
|  | **Middle** | -0.23 (-0.35, -0.11) | <0.001 |
|  | **Lowest** | -0.36 (-0.48, -0.23) | <0.001 |
|  | ***P* *for trend*** | <0.001 |  |
| **Mental status** |  |  |  |
|  | **Highest** | Reference |  |
|  | **Middle** | -0.18 (-0.36, -0.01) | 0.045 |
|  | **Lowest** | -0.35 (-0.54, -0.17) | <0.001 |
|  | ***P* *for trend*** | <0.001 |  |
| **Global cognition** |  |  |  |
|  | **Highest** | Reference |  |
|  | **Middle** | -0.40 (-0.65, -0.15) | 0.002 |
|  | **Lowest** | -0.65 (-0.91, -0.39) | <0.001 |
|  | ***P* *for trend*** | <0.001 |  |

^a^ Adjusted for age, sex, education, BMI, smoking, alcohol consumption, hypertension, diabetes, stroke, heart disease, lung disease, and follow-up time. Abbreviations: CIs, confidence intervals; BMI, body mass index.

**Supplementary Table 4.** The association between walking speed and change in cognitive function over time in the CHARLS with multiple imputation for data missing.

| **Cognitive function** | **Walking speed** | ***β* (95% CIs) ^a^** | ***P*** |
| --- | --- | --- | --- |
| **Episodic memory** |  |  |  |
|  | **Highest*time** | Reference |  |
|  | **Middle*time** | -0.04(-0.07, -0.01) | 0.029 |
|  | **Lowest*time** | -0.06(-0.09, -0.02) | 0.001 |
|  | ***P* *for trend*** | 0.001 |  |
| **Mental status** |  |  |  |
|  | **Highest*time** | Reference |  |
|  | **Middle*time** | -0.05(-0.09, -0.01) | 0.035 |
|  | **Lowest*time** | -0.07(-0.12, -0.03) | 0.001 |
|  | ***P* *for trend*** | 0.043 |  |
| **Global cognition** |  |  |  |
|  | **Highest*time** | Reference |  |
|  | **Middle*time** | -0.06 (-0.13, -0.00) | 0.050 |
|  | **Lowest*time** | -0.13 (-0.20, -0.06) | <0.001 |
|  | ***P* *for trend*** | 0.037 |  |

^a^ Adjusted for age, sex, education, BMI, smoking, alcohol consumption, hypertension, diabetes, stroke, heart disease, lung disease, and follow-up time. Abbreviations: CIs, confidence intervals; BMI, body mass index.

**Supplementary Table 5.** The association between baseline walking speed and subsequent cognitive functions by sex in the CHARLS.

| **Cognitive Functions** | **Walking speed** | **Female** | | **Male** | | ***P value for interaction*** |
| --- | --- | --- | --- | --- | --- | --- |
|  |  | β (95% CIs) ^a^ | *P* | β (95% CIs) ^a^ | *P* |  |
| **Episodic memory** | **Highest** | Reference |  | Reference |  |  |
|  | **Middle** | -0.21(-0.35, -0.08) | 0.001 | -0.23(-0.35, -0.11) | <0.001 | 0.995 |
|  | **Lowest** | -0.35(-0.48, -0.22) | <0.001 | -0.38(-0.51, -0.25) | <0.001 | 0.915 |
|  | ***P* *for trend*** | <0.001 |  | <0.001 |  |  |
| **Mental status** | **Highest** | Reference |  | Reference |  |  |
|  | **Middle** | -0.27(-0.51, -0.03) | 0.027 | -0.14(-0.32, 0.05) | 0.142 | 0.125 |
|  | **Lowest** | -0.53(-0.77, -0.29) | <0.001 | -0.38(-0.57, -0.18) | <0.001 | 0.046 |
|  | ***P* *for trend*** | <0.001 |  | <0.001 |  |  |
| **Global cognition** | **Highest** | Reference |  | Reference |  |  |
|  | **Middle** | -0.58(-0.90, -0.25) | 0.001 | -0.35(-0.61, -0.09) | 0.008 | 0.114 |
|  | **Lowest** | -0.90(-1.23, -0.57) | <0.001 | -0.75(-1.02, -0.47) | <0.001 | 0.142 |
|  | ***P* *for trend*** | <0.001 |  | <0.001 |  |  |

^a^ Adjusted for age, sex, education, BMI, smoking, alcohol consumption, hypertension, diabetes, stroke, heart disease, lung disease and follow-up time. Abbreviations: CIs, confidence intervals; BMI, body mass index.

**Supplementary Table 6.** The association between baseline walking speed and subsequent cognitive functions by education in the CHARLS.

| **Cognitive Functions** | **Walking speed** | **Primary school and below** | | **Middle school and above** | | ***P value for interaction*** |
| --- | --- | --- | --- | --- | --- | --- |
|  |  | β (95% CIs) ^a^ | *P* | β (95% CIs) ^a^ | *P* |  |
| **Episodic memory** | **Highest** | Reference |  | Reference |  |  |
|  | **Middle** | -0.28(-0.39, -0.18) | <0.001 | -0.14(-0.35, 0.07) | 0.201 | 0.184 |
|  | **Lowest** | -0.42(-0.53, -0.32) | <0.001 | -0.38(-0.62, -0.16) | 0.001 | 0.810 |
|  | ***P* *for trend*** | <0.001 |  | 0.001 |  |  |
| **Mental status** | **Highest** | Reference |  | Reference |  |  |
|  | **Middle** | -0.40(-0.61, -0.20) | <0.001 | -0.18(-0.39, 0.03) | 0.099 | 0.167 |
|  | **Lowest** | -0.72(-0.93, -0.50) | <0.001 | -0.32(-0.56, -0.09) | 0.006 | 0.033 |
|  | ***P* *for trend*** | <0.001 |  | 0.005 |  |  |
| **Global cognition** | **Highest** | Reference |  | Reference |  |  |
|  | **Middle** | -0.72(-0.99, -0.44) | <0.001 | -0.32(-0.68, 0.04) | 0.008 | 0.082 |
|  | **Lowest** | -1.13(-1.41, -0.85) | <0.001 | -0.69(-1.08, -0.30) | 0.001 | 0.069 |
|  | ***P* *for trend*** | <0.001 |  | 0.001 |  |  |

^a^ Adjusted for age, sex, education, BMI, smoking, alcohol consumption, hypertension, diabetes, stroke, heart disease, lung disease, and follow-up time. Abbreviations: CIs, confidence intervals; BMI, body mass index.

**Supplementary Table 7.** The association between motoric cognitive risk syndrome (MCR) and cognitive function at baseline in the CHARLS.

| **Cognitive function** | **MCR** | **β (95% CIs) ^a^** | ***P*** |
| --- | --- | --- | --- |
| **Episodic memory** | **No** | Reference |  |
|  | **Yes** | -0.24 (-0.37, -0.11) | <0.001 |
| **Mental status** | **No** | Reference |  |
|  | **Yes** | -0.45 (-0.66, -0.24) | <0.001 |
| **Global cognition** | **No** | Reference |  |
|  | **Yes** | -0.71 (-1.00, -0.43) | <0.001 |

^a^ Adjusted for age, sex, education, BMI, smoking, alcohol consumption, hypertension, diabetes, stroke, heart disease, lung disease, and follow-up time. Abbreviations: CIs, confidence intervals; BMI, body mass index.

**Supplementary Table 8.** The association between motoric cognitive risk syndrome (MCR) and change in cognitive function over time in the CHARLS with multiple imputation for data missing.

| **Cognitive function** | **MCR** | ***β* (95% CIs) ^a^** | ***P*** |
| --- | --- | --- | --- |
| **Episodic memory** | **No*time** | Reference |  |
|  | **Yes*time** | -0.03(-0.06, 0.01) | 0.074 |
| **Mental status** | **No*time** | Reference |  |
|  | **Yes*time** | -0.01(-0.05, 0.04) | 0.748 |
| **Global cognition** | **No*time** | Reference |  |
|  | **Yes*time** | -0.05 (-0.11, 0.02) | 0.163 |

^a^ Adjusted for age, sex, education, BMI, smoking, alcohol consumption, hypertension, diabetes, stroke, heart disease, lung disease, and follow-up time. Abbreviations: CIs, confidence intervals; BMI, body mass index.
